# The dual model of perfectionism and depression among Chinese University students

**DOI:** 10.4102/sajpsychiatry.v23i0.1025

**Published:** 2017-02-24

**Authors:** Yefei Wang, Bin Zhang

**Affiliations:** 1 Department of Psychiatry and Mental Health Institute, Second Xiangya Hospital, China; 2 Department of Applied Psychology, Hunan University of Chinese Medicine, China

## Abstract

The dual model of perfectionism was adopted to explore the influence of adaptive and maladaptive perfectionism on depression in college students. The results support the dual process model of perfectionism in Chinese undergraduates. A sample of 206 Chinese undergraduates completed measures of perfectionism, General Self-efficacy Measure, Beck Depression Inventory, State Anxiety Inventory, Positive and Negative Affect Scale (Time 1) and Beck Depression Inventory 4 months later (Time 2). Exploratory and confirmatory factor analysis revealed that the three-factor model of perfectionism with dimensions of maladaptive perfectionism, adaptive perfectionism and order factor fit the date well. Partial correlations analyses revealed that maladaptive perfectionism was related to psychopathology, whereas adaptive perfectionism was more closely correlated with positive features of mental health. In cross-sectional analyses, the discrepancy which measures the perceived difference between the standards one has set for one’s own behaviour and actual performance and the socially prescribed perfectionism subscales of maladaptive perfectionism could significantly predict baseline depressive symptoms. However, after controlling for the initial scores of depression, none of the perfectionism subscales significantly predicted the change in depression across a 4-month lag. Distinguishing adaptive and maladaptive aspects of perfectionism may be beneficial to understanding the influence of perfectionism on depression.

## Introduction

### Dual process model of perfectionism

Perfectionism is commonly defined as the tendency to set excessively high standards for performance accompanied by tendencies for overly critical evaluations of one’s behavior.^1^ Evidence has confirmed that adaptive (positive striving) and maladaptive (maladaptive evaluation concerns) perfectionism can be distinguished.^1^ Slade and Owens^2^ explicated a dual process model of perfectionism which was based on underlying functional differences. In this model, adaptive perfectionists were underlined by positive reinforcement, whereas maladaptive perfectionists were strengthened by negative reinforcement. Accordingly, adaptive perfectionists tend to set realistic rather than unreachable standards and emphasise achieving success rather than avoiding failure. On the contrary, maladaptive perfectionists seek to avoid or escape personal failure, tend to set unrealistically high standards and are driven by a fear of failure.^3^ Several lines of evidence support the conceptual, psychometric and practical importance of the dual model of perfectionism.^1,2^ Suddarth and Slaney^4^ conducted a factor analysis using three measures of perfectionism, The Hewitt and Flett Multidimensional Perfectionism Scale (HMPS), The Frost Multidimensional Perfectionism Scale (FMPS) and the Revised Almost Perfect Scale (APS-R) and found three factors which they labelled ‘maladaptive perfectionism’, ‘adaptive perfectionism’ and ‘orderliness’. A number of studies demonstrated that maladaptive perfectionism dimensions are strongly associated with negative outcomes, such as anxiety, depression and suicidal ideation,^5,6^ whereas adaptive perfectionism dimensions have shown positive relationships with positive outcomes such as positive affect (PA), academic achievement and life satisfaction.^7,8^

In recent years, the emergence of valid and reliable perfectionism scales has contributed to an increased understanding of the association between perfectionism and depression. However, it has been widely acknowledged that perfectionism is a multidimensional construct. Three popular measures assessing multiple dimensions of perfectionism have been developed.^9,10^ All of these measures are called the ‘Multidimensional Perfectionism Scale’, but they were developed by different conceptual frameworks. The FMPS^9^ assessed six dimensions which were as follows: concern over mistakes (CM), personal standards (PS), parental criticism (PC), parental expectations (PE), doubts about actions (DA) and organisation (O). The HMPS ^10^ measured self-oriented perfectionism (SOP), socially prescribed perfectionism (SPP) and other-oriented perfectionism (OOP). Slaney and colleagues^11,12^ developed the APS-R and argued that major constructs of perfectionism should include factors such as high personal standards (HS), perceived discrepancy between standards and performance (Dis) and preferences for order and organisation.

Mobley et al.^13^ noted that most of the published studies on perfectionism have focused on samples from Western universities. Additional studies on perfectionism investigating its relevance for diverse ethnic, racial and cultural groups are clearly needed. It makes means to examine if the three perfectionism scales (FMPS, HMPS and APS-R) converge into the same three dimensions (adaptive, maladaptive and organisation/order) in Chinese university students.

### Perfectionism and depression

Numerous studies using cross-sectional approaches in a variety of samples have found a medium to large correlation between CM, DA, and SPP and depressive symptoms, and a small positive correlation between SOP and depressive symptoms.^14,15^ In one study with a longitudinal design, SPP predicted an increase in levels of depressive symptoms over a 4-month period.^16^ Results of multiple regression analyses indicated that HS scores were negative predictors of depression levels, whereas discrepancy scores were positive predictors.^17^ Three perfectionism measures were used to investigate the relationship between perfectionism and depression, but as yet, no known study has compared these subscales to determine their relative efficacy in predicting depression levels. Most longitudinal studies regarding perfectionism and depression included only one single perfectionism scale. HMPS has been used in a series of studies to assess the change in depression scores over time with longitudinal analyses.^18,19^ There has been no published research examining the predictive efficacy of the three measures to the change in depression levels.

### The present study

The present study had several objectives. First, the main purpose was to provide evidence for the dual process model in Chinese students. It was hypothesised that the three perfectionism scales (FMPS, HMPS and APS-R) converge into the same three dimensions (adaptive, maladaptive and organisation/order) and maladaptive perfectionism would correlate positively with negative outcomes while adaptive perfectionism would correlate positively with positive outcomes. Second, a cross-sectional analysis was conducted to explore the relative importance of the different subscales in the dual process model. It was expected that subscales measuring maladaptive perfectionism would positively predict depression levels, whereas subscales of adaptive characteristics would negatively predict depression scores. Third, this study included a longitudinal analysis to assess the change in depression levels over time. It was also predicted that maladaptive dimensions of perfectionism would be worse off over time, accompanied with increased level of depression at Time 2, while adaptive dimensions of perfectionism would protect against increased level of depression at Time 2 after controlling for the depression baseline levels.

## Method

### Participants

Participants were recruited from an introductory psychology course in Changsha, Hunan (China), at Central South University. A total of 236 participants (106 men) participated in the Time 1 assessment. Completed Time 1 and Time 2 data were available for 206 undergraduate students (97 men). The average age of the participants at Time 1 was 20.16 years (s.d. = 1.99). There were 80 freshmen, 105 sophomores and 21 juniors.

### Measures

Hewitt and Flett Multidimensional Perfectionism Scale (HMPS; Hewitt and Flett)^10^ *–* This 45-item scale is composed of three subscales: self-oriented (the setting of excessively high standards for oneself), other-oriented (the perception that others hold excessively high standards for oneself) and socially prescribed perfectionism (holding unrealistic standards of performance or behaviour for significant others). Items are responded to using a scale of 1 = *disagree* to 7 = *agree*. The authors reported reliability coefficients of 0.88, 0.74 and 0.81 for SOP, OOP and SPP, respectively.^2^ The Chinese version of Multidimensional Perfectionism Scale, which consists of three subscales SOP (14 items), OOP (9 items) and SPP (10 items), was used in the present study.^20^ The Chinese version of the HMPS has been investigated in a number of studies and has shown sound psychometric characteristics.^21^ In this study, the Cronbach’s alpha was 0.87 for SOP, 0.69 for OOP and 0.74 for SPP.

Frost Multidimensional Perfectionism Scale (FMPS; Frost et al.)^9^ *–* FMPS contains 35 self-reported items that are scored as six subscales: CM, PS, PE, PC, DA, and organisation (OR). Items are responded to using a scale of 1 = *disagree strongly* to 5 = *agree strongly*. Several studies revealed Cronbach’s coefficient alphas for the FMPS subscales ranged from 0.70 to 0.93.^15^ The Chinese version of MPS was translated by Zi and Zhou.^22^ Their factor analysis extracted five of the original six factors of FMPS. PC did not emerge as a dimension. The five subscales were shown to have satisfactory internal consistencies with Cronbach’s alpha and test–retest reliability. Chinese FMPS was valid and reliable for use among college students. The Chinese version of FMPS is composed of five subscales: CM (6 items), DA (4 items), PS (6 items), PE (5 items) and OR (6 items). In this study, the Cronbach’s alpha was 0.84 for CM, 0.72 for DA, 0.75 for PS, 0.84 for PE and 0.83 for organisation.

Almost Perfect Scale-Revised (APS-R; Slaney et al.)^11,12^ – APS-R is a self-report measure that measures three dimensions of perfectionism: high standards, order and discrepancy. Twenty-three items are rated on a seven-point Likert scale (1 = *strongly disagree* to 7 = *strongly agree*). Slaney et al.^11^ reported Cronbach’s alphas on high standards, order and discrepancy to be 0.85, 0.86 and 0.92, respectively.^3^ The Chinese version of the APS-R^23^ was used in the present study. Acceptable internal consistency estimates of the Chinese APS-R have been reported.^23^ In this study, the Cronbach’s alpha was 0.79 for high standards scale, 0.72 for order scale and 0.88 for discrepancy scale.

Rosenberg Self-Esteem Scale (SES; Rosenberg) *–* The SES is a widely used 10-item scale designed to assess individuals’ global self-esteem.^24^ The psychometric properties of the SES have been reported.^25^ Each item is rated on a four-point Likert scale (1= *strongly disagree* to 4 = *strongly agree*). A Chinese translation of the SES was used in this study. In this study, the Cronbach’s alpha was 0.76 for the SES scale.

General Self-Efficacy Scale (GSES; Zhang and Schwarzer) *–* Zhang and Schwarzer^26^ developed the Chinese GSES on the basis of Bandura’s conceptualisation of his early theory of self-efficacy. The GSES is a 10-item, four-point Likert-format instrument (1 = *not at* *all true* to 4 = *exactly true*), with higher scores indicating greater self-efficacy. A summary of the responses to all 10 items yield the final composite score with a range from 10 to 40. The scale has been validated in previous studies. In this study, the Cronbach’s alpha was 0.86 for the Beck Depression Inventory (BDI; Beck et al.). BDI is a 21-item questionnaire assessing cognitive, somatic, and behavioural indices of depression. Each item is rated on a four-point Likert scale (0–3).^27^ The scale is a widely used measure of depressive symptoms in various populations, including the Chinese.^28^ The Chinese BDI was found to have satisfactory internal consistency (α = 0.86).^29^ In this study, the Cronbach’s alpha was 0.82 for the BDI scale. Participants completed the BDI at Time 1 and Time 2.

The State Anxiety Inventory (SAI; Spielberger) *–* The 20-item SAI is derived from the State-Trait Anxiety Inventory. Each item is rated on a four-point Likert scale (1 = *almost never* to 4 = *almost always*). The SAI has satisfactory internal consistency with Cronbach’s alpha and test–retest reliability.^30^ The Chinese SAI has been reported as satisfactorily valid and reliable.^31^ In this study, the Cronbach’s alpha was 0.90 for the SAI scale.

Positive and Negative Affect Scale (PANAS; Watson et al.) *–* This scale assesses the distinct dimension of trait positive and negative affect. Items are rated on a five-point Likert-type instrument with two subscales; positive affect (PA) and negative affect (NA). Psychometric data have shown excellent internal consistency, test-retest reliability and satisfactory validity for this measure.^32^ The Chinese PANAS has been reported as satisfactorily valid and reliable.^33^ In the present study, the Cronbach’s alpha was 0.84 for the PA scale and 0.84 for NA scale.

## Ethical considerations

The research was approved by the Ethics Committee of the Central South University. The participants completed the first set of questionnaires in the beginning of the semester (September – October) during school hours (Time 1 [T1]). After 4 months, at the end of the semester (January – February), participants completed the second set of questionnaire (Time2 [T2]). The time lag was about 4 months in this study. Students participated in the study on a voluntary and anonymous basis. The investigators assigned and identified the participants by code numbers.

## Results

### Exploratory and confirmatory factor analysis

To confirm the adequacy of the dual model, the three perfectionism scales were subjected to factor analysis, using an orthogonal, VARIMAX rotation. The result was an unambiguous three-factor solution with the factor discrepancy (the degree to which the respondents perceive themselves as failing to meet their standards for performance), CM, SPP (the perception that others hold excessively high standards for oneself) and parental expectation loading on factor 1 (maladaptive perfectionism); SOP (the setting of excessively high standards for oneself), high standards and OOP (holding unrealistic standards of performance or behaviour for significant others) loading on factor 2 (adaptive perfectionism); and organisation or order (a preference for neatness and orderliness) loading on factor 3 (orderliness). This result was consistent with previously reported factor analyses of the perfectionism subscales.^4^ Additionally, confirmatory factor analysis was performed using Amos 5.0 software to determine if the three perfectionism scales were best explained as three-factor models. All subscales loaded significantly on their respective factors, with loadings ranging from 0.39 to 0.91, except that OOP subscale loading was 0.20 (*p* > 0.05). The OOP subscale has not been demonstrated to be relevant to depression in previous studies.^17^ Therefore, OOP was excluded from further analyses. The final model fits well (GFI = 0.92, CFI = 0.93, NFI = 0.90, RMSEA = 0.073).^34^ In this study, only the two facets of perfectionism – adaptive and maladaptive perfectionism – were used.

### Descriptive statistics and correlations

Table 1 shows the correlations among all study measures as well as means and standard deviations. Correlations were in the expected directions. Beck Depression Inventory time 1 (BDI T1) showed a large correlation with Beck Depression Inventory time 1 (BDI T2) (*r* = 0.63, *p* < 0.001). Both adaptive and maladaptive perfectionism were positively correlated with SAI (state anxiety) as well as BDI (depression) T1, although maladaptive perfectionism showed a stronger association with SAI and BDI T1 than adaptive perfectionism. Maladaptive perfectionism was significantly correlated with both BDI T1 (*r* = 0.59, *p* < 0.001) and T2 (*r* = 0.49, *p* < 0.001), whereas adaptive perfectionism showed a small correlation with BDI T1 only (*r* = 0.15, *p* < 0.05). Adaptive perfectionism was positively correlated with PA (*r* = 0.21, *p* < 0.01), whereas maladaptive perfectionism was negatively correlated with PA (*r* = -0.21, *p* < 0.01). Only maladaptive perfectionism was significantly associated with self-esteem and general self-efficacy (*r* = -0.53 and *r* = -0.20, *p* < 0.01, respectively), as well as NA (*r* = 0.38, *p* < 0.001). The correlation between adaptive and maladaptive perfectionism was positive (*r* = 0.39, *p* < 0.001). The results indicated that maladaptive perfectionism also shares the common variance of adaptive perfectionism. Therefore, this overlap would lead to the positive relationship in the two aspects of perfectionism.^35^

**TABLE 1:** Correlations and descriptive statistics (*N* = 206).

Variable	1	2	3	4	5	6	7	8	9
1. Adaptive									
2. Maladaptive	0.39***								
3. SAI	0.15*	0.48***							
4. NA	0.12	0.38***	0.61***						
5. PA	0.21**	-0.21**	-0.41***	-0.02					
6. SES	0.04	-0.53***	-0.53***	-0.39***	0.42***				
7. GSES	0.12	-0.20**	-0.30***	-0.15*	0.37***	0.47***			
8. BDI T1	0.15*	0.59***	0.64***	0.49***	-0.40***	-0.49***	-0.18*		
9. BDI T2	0.09	0.49***	0.41***	0.29***	-0.19**	-0.31***	-0.15*	0.63***	
M	107.71	124.92	40.68	22.64	31.02	28.32	22.68	9.30	8.54
s.d.	18.93	22.98	8.70	6.14	5.58	3.03	4.53	6.65	7.00

Adaptive, adaptive perfectionism; Maladaptive, maladaptive perfectionism; SAI, state-anxiety inventory; NA, negative affect; PA, positive affect; SES, self-esteem scale; GSES, general self-efficacy scale; BDI T1, Beck Depression Inventory time 1; BDI T2, Beck Depression Inventory time 2.

* *p* < 0.05;

** *p* < 0.01;

*** *p* < 0.001

To explore the unique relationship of each aspect of perfectionism (maladaptive perfectionism; adaptive perfectionism) with the measures of mental health, partial correlations were calculated (see Table 2). The results indicated that the association of maladaptive perfectionism to these measures remained consistent after controlling for adaptive perfectionism. Adaptive perfectionism remained uncorrelated with NA and significantly positively correlated with PA after controlling for maladaptive perfectionism. Once maladaptive perfectionism was covaried, there were two kinds of changes to the pattern of correlations for adaptive perfectionism and mental measures. First, the non-significant correlation between adaptive perfectionism and self-esteem, as well as general self-efficacy, became significant after controlling for maladaptive perfectionism. Second, adaptive perfectionism was no longer positively correlated with both SAI and BDI T1.

**TABLE 2:** Correlations (and partial correlations) between the dimensions of perfectionism and mental health.

Variable	SAI	NA	PA	SES	GSES	BDI T1
Adaptive	0.15*	0.12	0.21**	0.04	0.12	0.15*
Controlling for maladaptive	-0.04	-0.04	0.33***	0.32***	0.22**	-0.12
Maladaptive	0.48***	0.38***	-0.21**	-0.53***	-0.20**	0.59***
Controlling for adaptive	0.46***	0.36***	-0.34***	-0.59***	-0.27***	0.58***

SAI, state-anxiety inventory; NA, negative affect; PA, positive affect; SES, self-esteem scale; GSES, general self-efficacy scale; BDI T1, Beck Depression Inventory time 1.

### Specificity of perfectionism subscales in predicting depression in cross-sectional analyses

Another purpose of the study was to examine the relative importance of the different perfectionism dimensions, as assessed by the HMPS (SOP, SPP), the FMPS (CM, PS, PE, DA), the APS-R (HS, Dis), in explaining variations in depression levels. Because there was no rationale for the primacy of some measures of perfectionism over others, the relevant subscale scores from each of the three perfectionism scales were entered simultaneously in cross-sectional multiple regression equation, predicting depressive symptoms (see Table 3). Multicollinearity statistics revealed no substantial concerns in these analyses (variance inflation factors [VIF] ranged from 1.07 to 2.50 and tolerances ranged from 0.40 to 0.94). Values for VIF exceeded 10; tolerance values less than 0.10 are generally regarded as concerns with regard to multicollinearity. In the prediction of the BDI T1 scores, the perfectionism subscales combined to account for 38.1% of the variance, *>F*>(10, 193) = 11.88, *p* < 0.001. In this analysis, only the DIS (β = 0.372, *p* < 0.001) and the SPP (β = 0.264, *p* < 0.01) subscales emerged as significant predictors. The current results also indicated that only the maladaptive aspects of perfectionism could significantly predict the depressive symptoms, whereas the adaptive aspect of perfectionism did not.

**TABLE 3:** Regression of BDI T1 on perfectionism (cross-sectional multiple regression analyses).

Variable	BDI T1	
	*β*	∆R2
**Step 1**		0.009
Gender	-0.004	
Age	0.073	
**Step 2**		0.372***
**(Maladaptive perfectionism)**		
Dis	0.372***	
SPP	0.264**	
CM	0.134	
DA	0.013	
PE	0.072	
**(Adaptive perfectionism)**		
HS	-0.166	
SOP	0.091	
PS	-0.109	

Gender coded as male = 1, female = 2. Dis, discrepancy; SPP, socially prescribed perfectionism; CM, concern over mistakes; DA, doubts about actions; PE, parental expectations; HS, high standards; SOP, self-oriented perfectionism; PS, personal standards.

### Hierarchical multiple regression for perfectionism and depression in longitudinal analyses

Hierarchical regression analysis was used to determine whether maladaptive and adaptive perfectionism (measured at T1) would predict depression level at T2. Demographic variables (gender, age) were controlled in step 1. Given the high comorbidity rate between anxiety and depression, the anxiety and depression levels at T1 were controlled in step 2. The subscales of perfectionism were entered as predictors in step 3 (see Table 4). Multicollinearity statistics revealed no substantial concerns in these analyses (VIFs ranged from 1.02 to 2.85 and tolerances ranged from 0.35 to 0.98). However, the results indicated that none of perfectionism subscales was a significant predictor of BDI T2. In this hierarchical regression equation, only BDI T1 (β = 0.543, *p* < 0.001) could significantly predict BDI T2.

**TABLE 4:** Regression of BDI T2 on perfectionism T1 after controlling for BDI T1 (longitudinal analyses).

Variable	BDI T2	
	*β*	∆R2
***Step* 1**		0.012
Gender	0.057	
Age	0.033	
**Step 2**		0.390***
BDI T1	0.543***	
SAI	-0.032	
**Step 3**		0.030
**(Maladaptive perfectionism)**		
Dis	0.121	
SPP	0.016	
CM	0.026	
DA	0.013	
PE	0.045	
**(Adaptive perfectionism)**		
HS	-0.046	
SOP	-0.083	
PS	0.078	

BDI T2, Beck Depression Inventory time 2; BDI T1, Beck Depression Inventory time 1; SAI, state-anxiety inventory; Dis, discrepancy; SPP, socially prescribed perfectionism; CM, concern over mistakes; DA, doubts about actions; PE, parental expectations; NA, negative affect; PA, positive affect; HS, high personal standards; SOP, self-oriented perfectionism; PS, personal standards.

## Discussion

Consistent with the findings by Suddarth and Slaney,^4^ the present research using EFA and CFA supported the contention that perfectionism may be best explained as a three-factor construct using the dimensions of adaptive perfectionism, maladaptive perfectionism and orderliness.

As hypothesised, both zero-order and partial correlations revealed a positive association of maladaptive scores and a number of measures of psychopathology (state anxiety, NA and depression). In addition, maladaptive perfectionism was negatively correlated with positive outcomes (PA, self-esteem and general self-efficacy). Adaptive perfectionism was positively correlated with positive outcomes, whereas it was not correlated with psychopathology measures after maladaptive perfectionism scores were controlled. Overall, the data are consistent with the contention that maladaptive perfectionism, including setting unrealistically high standards and the fear of failure, is mostly closely associated with psychopathology, whereas adaptive perfectionism involving the setting of realistic goals and feelings of achievement satisfaction are often closely correlated with positive outcomes. Thus, the results support the dual process model and demonstrated that it is improper to portray perfectionism in singularly maladaptive or pathological terms.

In the cross-sectional regression analyses, results indicated that only the subscales of maladaptive perfectionism (discrepancy of APS-R, SPP of FMPS), but none of subscales from adaptive perfectionism, could predict depression levels. The current findings also revealed that the discrepancy and SPP subscales were more efficacious predictors of depressive symptoms, compared with other subscales. These findings are consistent with other research findings on perfectionism and depression.^16^ In the APS-R, discrepancy was defined as the perceived difference between the standards one has set for one’s own behaviour and actual performance. Slaney et al.^11^ have argued that the factor of discrepancy should be a central indicator of maladaptive perfectionism. Maladaptive perfectionists viewed temporary failure as a failure forever and were not satisfied with the discrepancy between the goals they set and the performance they attained.^36^ SPP was the only HMPS dimension to emerge as a significant predictor of depressive symptoms. Socially prescribed perfectionists believe that others hold unrealistically high standards of them, evaluate them critically and exert excessive pressure on socially prescribed perfectionists to be ‘perfect’.^10^ Since these standards are perceived as being excessive and uncontrollable, socially prescribed perfectionists may be subjected to depression and anxiety resulting from an inability to attain such standards. This is consistent with other studies that indicated more maladaptive aspects of perfectionism are related to depression.^37^ The results of this study have implications for the professional practice of school counselling. For example, when treating a Chinese client who has both perfectionistic characteristics and depressive symptoms, counsellors need to discern between these two aspects of perfectionism. Treatment would help clients increase satisfaction with their performance and reduce depression by lowering the discrepancy of APS-R and SPP of HMPS scores. Helping young clients discern between their goals and reality, and between their expectations and those of their parents, may be helpful.

In the longitudinal regression analyses, results indicated that after controlling for depression and anxiety at T1, neither subscales of maladaptive perfectionism nor those of adaptive perfectionism were significant predictors of BDI T2. This finding is inconsistent with previous research that SPP could predict depression and increased levels of depressive symptoms over time in patients sample.^17^ Two possible explanations may account for the lack of a link between perfectionism and depression. Firstly, the 4-month test–retest period is too short to detect the changes of depression levels. It is necessary to extend the period of longitudinal observation in further investigation. Secondly, the sample of the current study only included Chinese university students (non-clinical sample), but not depression patients (clinical sample). Additionally, most participants were freshmen and sophomores, and they might not experience extreme pressure from study and employment. However, these explanations need to be further investigated in future studies, especially personal predisposing factors should be taken into consideration when investigating the link between pressure and depression.

## Limitations and conclusion

A few limitations of the study need to be stated. Firstly, the study relied on a convenient, non-clinical sample. Therefore, appropriate caution should be exercised in generalising the findings. It would be necessary to confirm whether the findings can be replicated in a clinical sample. Secondly, the data collected in this study came from self-report measures, and thus, the method of variance might inflate the relationships between variables. Future studies may benefit from the incorporation of interview-based assessment and significant-other reports. Thirdly, the timespan with a 4-month lag between T1 and T2 in the longitudinal study was relatively short; a longer time period is recommended in future studies. In summary, the study provided preliminary evidence for the dual process model in Chinese undergraduates. The study demonstrated that maladaptive perfectionism was related to psychopathology, whereas adaptive perfectionism was more closely correlated with positive features of mental health. Distinguishing adaptive and maladaptive aspects of perfectionism, the relationship between perfectionism and depression with cross-sectional and longitudinal analyses was explored. Specifically, the results indicated that only the subscales of maladaptive perfectionism (Dis of APS-R and SPP of HMPS) served as the significant and positive predictors of the depression levels in the current cross-sectional analyses, while the longitudinal analysis failed to detect the change in depression scores over time.
